# The RNA-binding Protein TDP-43 Selectively Disrupts MicroRNA-1/206 Incorporation into the RNA-induced Silencing Complex*^♦^

**DOI:** 10.1074/jbc.M114.561902

**Published:** 2014-04-09

**Authors:** Isabelle N. King, Valeria Yartseva, Donaldo Salas, Abhishek Kumar, Amy Heidersbach, D. Michael Ando, Nancy R. Stallings, Jeffrey L. Elliott, Deepak Srivastava, Kathryn N. Ivey

**Affiliations:** From the ‡ Gladstone Institute of Cardiovascular Disease and; ‖ Gladstone Institute of Neurological Disease, San Francisco, California 94158,; the Departments of § Pediatrics,; ‡‡ Biochemistry and Biophysics, and; ¶ Biomedical Sciences, Graduate Program, University of California, San Francisco, California 94158, and; the ** Department of Neurology and Neurotherapeutics, the University of Texas Southwestern Medical Center, Dallas, Texas 75235

**Keywords:** Cardiac muscle, Gene Regulation, MicroRNA, RNA-binding Protein, RNA-Protein Interaction, Skeletal Muscle

## Abstract

MicroRNA (miRNA) maturation is regulated by interaction of particular miRNA precursors with specific RNA-binding proteins. Following their biogenesis, mature miRNAs are incorporated into the RNA-induced silencing complex (RISC) where they interact with mRNAs to negatively regulate protein production. However, little is known about how mature miRNAs are regulated at the level of their activity. To address this, we screened for proteins differentially bound to the mature form of the *miR-1* or *miR-133* miRNA families. These muscle-enriched, co-transcribed miRNA pairs cooperate to suppress smooth muscle gene expression in the heart. However, they also have opposing roles, with the *miR-1* family, composed of *miR-1* and *miR-206*, promoting myogenic differentiation, whereas *miR-133* maintains the progenitor state. Here, we describe a physical interaction between TDP-43, an RNA-binding protein that forms aggregates in the neuromuscular disease, amyotrophic lateral sclerosis, and the *miR-1*, but not *miR-133*, family. Deficiency of the TDP-43 *Drosophila* ortholog enhanced *dmiR-1* activity *in vivo*. In mammalian cells, TDP-43 limited the activity of both *miR-1* and *miR-206*, but not the *miR-133* family, by disrupting their RISC association. Consistent with TDP-43 dampening *miR-1/206* activity, protein levels of the *miR-1/206* targets, IGF-1 and HDAC4, were elevated in TDP-43 transgenic mouse muscle. This occurred without corresponding *Igf-1* or *Hdac4* mRNA increases and despite higher *miR-1* and *miR-206* expression. Our findings reveal that TDP-43 negatively regulates the activity of the *miR-1* family of miRNAs by limiting their bioavailability for RISC loading and suggest a processing-independent mechanism for differential regulation of miRNA activity.

## Teaser

**Background:** Regulation of microRNA activity independent of processing and biogenesis has not been demonstrated.

**Results:** The RNA-binding protein, TDP-43, interacts with mature *miR-1*/*miR-206*, limiting their RNA-induced silencing complex (RISC) association and activity.

**Conclusion:** RNA-binding proteins can selectively control microRNA activity by disrupting RISC incorporation.

**Significance:** This is the first known microRNA-protein interaction that controls microRNA activity independent of processing.

## Introduction

MicroRNA (miRNA)^2^ biogenesis involves a series of processing steps, many of which are regulated by interaction of precursor forms of the miRNA with specific RNA-binding proteins. Of the over 1,000 miRNAs encoded in mammalian genomes, the majority are derived from primary microRNAs that encode two or more mature miRNAs within a common transcript. Although these polycistronic miRNAs share common transcriptional regulation, they often have varying, and sometimes even opposing, effects on cellular biology. How cells differentially regulate the activity of mature forms of co-transcribed miRNAs to achieve specific biological outcomes remains unknown. In particular, whether interaction of RNA-binding proteins with mature miRNAs regulates their activity has not been investigated.

**FIGURE 1.**
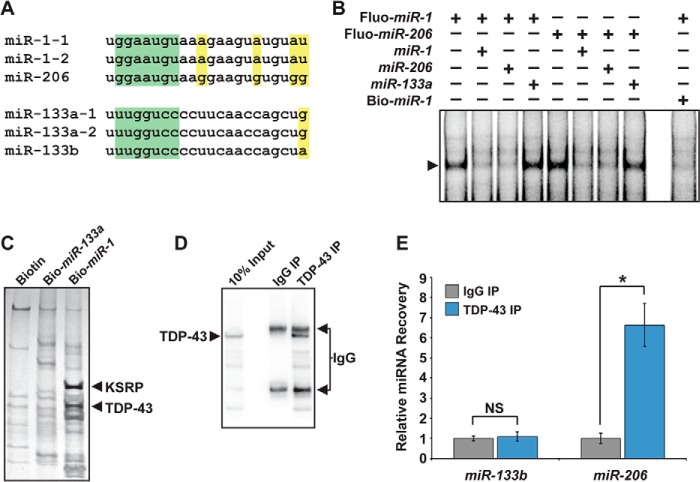
**TDP-43 interacts with the *miR-1/miR-206* family, but not *miR-133*.** *A*, sequence alignment of the *miR-1/miR-206* family or the *miR-133* family. miRNA seed sequence is highlighted in *green*. Residues that differ among family members are highlighted in *yellow. B*, EMSAs revealed an miRNA-protein complex (*arrow*) in undifferentiated C_2_C_12_ cell lysate that interacted with fluorescently labeled *miR-1* or *miR-206* probe (Fluo-*miR-1*, Fluo-*miR-206*). These interactions could be competed with unlabeled *miR-1* or *miR-206*, but not with unlabeled *miR-133a*. 5′-biotinylated *miR-1* (Bio-*miR-1*) also effectively competed for binding. *C*, eluates from negative control (*Biotin*), Bio-*miR-133a*, or Bio-*miR-1* pulldowns were run on denaturing gels. Proteins enriched in the Bio-*miR-1* pulldown lane were identified by mass spectrometry and included KSRP and TDP-43. *D*, Western analysis detecting TDP-43 protein following cross-linking and immunoprecipitation from C_2_C_12_ skeletal myoblasts using α-TDP-43 (*TDP-43 IP*), rabbit IgG (*IgG IP*), or 10% input showed efficient and specific recovery of TDP-43 protein. *E*, qRT-PCR to detect *miR-206* and *miR-133b* after TDP-43 cross-linking and immunoprecipitation performed in *D* revealed preferential interaction of TDP-43 with *miR-206 versus miR-133b*. Results were normalized to pulldown with IgG (*NS*, not significant; *, *p* < 0.05).

Muscle development and homeostasis are regulated, in part, by polycistronic microRNAs. The *miR-1* family, composed of *miR-1* and *miR-206*, whose mature sequences are nearly identical, and the *miR-133* family (1, 2) are highly conserved and are enriched in cardiac and skeletal muscle in species as distantly related as flies and humans (3–5) (see Fig. 1*A*). In mammals, up to three genomic loci encode bicistronic transcripts to produce *miR-133* and either *miR-1* or *miR-206*. In most higher vertebrates, the *miR-1/miR-133a* genomic locus was duplicated, with the expression of both loci being maintained in cardiac and skeletal muscle. A third locus encodes *miR-206* and *miR-133b* and is uniquely expressed in skeletal, not cardiac, muscle.

Targeted deletions of *miR-1* or *miR-133* in mice generally result in impaired cardiac development and function (6–11). Although *miR-1* and *miR-133* cooperate to repress smooth muscle gene expression in the heart (6, 7, 10, 11), *miR-1* promotes differentiation of striated muscle progenitors, whereas *miR-133* maintains the undifferentiated state *in vitro* (5, 12, 13). Deletion of one *miR-1* locus or both *miR-1* loci causes cardiac defects without a detectable skeletal muscle phenotype, likely due to the persistent expression of *miR-206* in skeletal muscle (7, 8, 11). Targeted deletion of *miR-206* alone does not disrupt muscle development or function in mice, but prevents efficient regeneration of neuromuscular junctions (NMJs) after acute nerve injury, leading to severe muscle loss (9). Deficiency of *miR-206* in the SOD-1 mouse model of amyotrophic lateral sclerosis (ALS) also accelerates disease progression, which is characterized by skeletal muscle atrophy from motor neuron degeneration and NMJ disruption (9). This hastening occurs although *miR-206* is not expressed in motor neurons, which are the primary cell type affected in ALS, and highlights the importance of bidirectional signaling between motor neurons and skeletal muscle for maintaining the NMJ. Similarly, in worms, muscle expression of *miR-1* is important for retrograde signaling to the motor neuron resulting in NMJ maintenance (14). Although evidence of *miR-1* or *miR-206* dysregulation in mouse models or human cases of ALS has not been reported, skeletal muscle up-regulation of the *miR-1/206* target, HDAC4, which inhibits muscle reinnervation, is positively correlated with ALS progression and severity (15).

The *miR-1* and *miR-133* family loci are under transcriptional control of key myogenic proteins including myogenin, MyoD, serum response factor (SRF), myocardin (MYOCD) (3, 4, 16), and myocyte-enhancing factor 2 (MEF-2) (17). Post-transcriptionally, several proteins regulate *miR-1* family biogenesis including the KH-type splicing regulatory protein (KSRP) (18), Muscleblind-like splicing regulator (MBNL1) (19), and RNA-binding protein LIN28 (19). These factors presumably control *miR-1* family levels in specific contexts. Given the extended half-life of miRNAs and the observations from deep-sequencing studies that the *miR-1* family accounts for up to half of accumulated miRNAs in cardiac and skeletal muscles (20, 21), directly controlling the activity of these critical myogenic regulators and their differential activity as compared with *miR-133* may be important to maintain muscle homeostasis. Although regulation of these bicistronic miRNAs has been studied at the level of post-transcriptional processing, proteins that differentially interact with and regulate the activity of these co-transcribed miRNAs have not been reported.

Here, we report that TDP-43, an RNA-binding protein that aggregates in individuals afflicted with ALS, physically associates with the mature form of the *miR-1/miR-206* family of miRNAs in muscle cells, but not with the co-transcribed *miR-133*. Our results demonstrate that TDP-43 negatively regulates the activity of *miR-1* and *miR-206* in muscle through a physical interaction that limits their bioavailability for RNA-induced silencing complex (RISC) loading and offer a mechanism by which mature miRNAs can be differentially regulated at the level of their activity. These findings also establish, for the first time, a mechanistic link between TDP-43 and the *miR-1/miR-206* family that may be an unappreciated component of ALS pathogenesis.

## EXPERIMENTAL PROCEDURES

### 

#### 

##### Electromobility Shift Assays

Cell extract (5 μg) was incubated with 1 pmol of fluor-conjugated miRNA (Integrated DNA Technologies) in 20 μl of 1× binding buffer (60 mm KCl, 10 mm HEPES, pH 7.6, 3 mm MgCl_2_, 5% glycerol, 1 mm DTT, 0.1 μg/μl tRNA, 5 μg/μl heparin) for 20 min at room temperature. Where indicated, 100 pmol of unlabeled or biotinylated miRNA competitor was added. Samples were separated on 6% polyacrylamide nondenaturing gel (without loading buffer in sample lanes) and imaged on a LI-COR Odyssey system.

##### RNA Pulldown

Undifferentiated C_2_C_12_ cells, grown in 10-cm dishes, were harvested in 1 ml of lysis buffer (10% glycerol, 20 mm Tris-HCl, pH 8.0, 0.2 mm EDTA, 0.1% Nonidet P-40, 0.5 m KCl) with fresh protease inhibitor (1 tablet/10 ml of lysis buffer, Complete protease inhibitor cocktail tablets, EDTA-free, Roche Applied Science). Lysate (30 mg) was precleared by incubating with streptavidin-conjugated Dynabeads (Life Technologies) on a rotator for 1 h at 4 °C. Lysate was incubated with 5′-end biotinylated miRNA (Integrated DNA Technologies) on a rotator overnight at 4 °C. Complexes were pulled down by incubation with streptavidin-conjugated Dynabeads on a rotator for 1 h at 4 °C. Beads were then washed extensively with lysis buffer, boiled in Laemmli buffer, separated on 4–12% gradient polyacrylamide gels (Bio-Rad), and stained with SilverQuest kit (Invitrogen).

##### Mass Spectrometry

Gel pieces at the appropriate molecular weight were isolated with a fresh razor blade and dehydrated by two 10-min washes with 25 mm ammonium bicarbonate, 70% acetonitrile. The disulfide bonds were reduced with 10 mm DTT in 25 mm ammonium bicarbonate for 45 min at 50 °C; reduced cysteines were alkylated by 50 mm iodoacetamide in 25 mm ammonium bicarbonate for 60 min at room temperature in the dark. Pieces were washed twice for 5 min with 25 mm ammonium bicarbonate, 70% acetonitrile. Proteins were trypsin-digested overnight at 37 °C. Supernatants were collected, each piece was incubated in 50% acetonitrile, 5% formic acid for 10 min, and supernatants were collected again. The total supernatant was dried on a SpeedVac and reconstituted in 0.1% formic acid. The samples were desalted using Zip-Tip C18 cartridge columns (Millipore) and run at the University of California San Francisco (UCSF) Mass Spectrometry Facility.

##### TDP-43 Cross-linking and Immunoprecipitation (CLIP)

TDP-43 CLIP was as described (22). Briefly, C_2_C_12_ cells were irradiated in a UV cross-linker once for 400 ml/cm^2^ and again for 200 mJ/cm^2^, harvested in 1× PXL (0.1% SDS, 0.5% Nonidet P-40, 0.5% deoxycholate, in 1× PBS) with protease inhibitors (1 tablet/10 ml of lysis buffer, Complete protease inhibitor cocktail tablets, EDTA-free, Roche Applied Science) and RNasin (250 units/ml; Promega), and treated with DNase for 5 min at 37 °C. Lysates were centrifuged at 10,000 × *g* at 4 °C for 10 min to remove cell debris and then precleared with protein A Dynabeads (Life Technologies) at 4 °C for 60 min. An input sample of each lysate was set aside for subsequent analysis. Each lysate was then divided and incubated with 5 μg/ml of either α-TDP-43 (Proteintech) or rabbit IgG (Millipore) on a rotator overnight at 4 °C. Complexes were pulled down by incubation with protein A Dynabeads on a rotator for 2 h at 4 °C. Beads were washed twice with 1× PXL, washed twice with 5× PXL (0.1% SDS, 0.5% Nonidet P-40, 0.5% deoxycholate, in 5× PBS), and boiled to reverse UV cross-linking. Co-precipitated RNAs were extracted and analyzed by quantitative RT-PCR (qRT-PCR).

##### Drosophila Studies

All flies were maintained on standard fly medium. The TDP-43^Q367X^ *Drosophila* line was generously provided by Fen-Biao Gao (University of Massachusetts Medical School) (23). All other lines were from the Bloomington Stock Center. Wild-type control flies were *W^1118^*. Transgene expression was achieved using the UAS-GAL4 system (*dpp*-GAL4, Bloomington Stock Center). Crosses were performed at 18 °C unless otherwise specified. *Drosophila* of the appropriate genotype were anesthetized with CO_2_ and then placed in isopropyl alcohol for 1 min and euthanized. Flies were allowed to air dry, and wings were removed and embedded into Canada Balsam (Sigma).

##### Luciferase Reporter Assays

C_2_C_12_ cells were maintained in DMEM, 10% FBS and transfected in triplicate with the indicated vectors, miRNA mimics (Life Technologies, *miR-1*: PM10660; *miR-206*: PM10409 or Pre-miR miRNA Precursor Negative Control #1), and siRNAs (Sigma, Mission siRNA Universal Negative Control #1: SIC001; siTDP-43: SAS1-Mm01-00198818) using Lipofectamine 2000 (Life Technologies). Total DNA/RNA was equivalent for each transfection condition. After 20 h, cells were harvested, and luciferase activity was measured in duplicate using the Dual-Luciferase reporter system (Promega) on a Victor 1420 multilabel counter (PerkinElmer). Luciferase activity was normalized to the activity of a co-transfected *Renilla* reporter.

##### AGO2 RNA Immunoprecipitation

C_2_C_12_ cells were maintained in DMEM, 10% FBS and transfected with the indicated siRNAs (Sigma, Mission siRNA Universal Negative Control #1: SIC001; siTDP-43: SAS1-Mm01-00198818) using Lipofectamine 2000 (Life Technologies). Argonaute2 (*AGO2*) RNA immunoprecipitation (IP) was performed as described (29). Cells were harvested in AGO2 lysis buffer (25 mm Tris-HCl, pH 8.0, 150 mm NaCl, 2 mm MgCl_2_, 0.5% Nonidet P-40, and 5 mm DTT) containing protease inhibitors (1 tablet/10 ml of lysis buffer, Complete protease inhibitor cocktail tablets, EDTA-free, Roche Applied Science) and RNasin (250 units/ml; Promega) after 24 h. Lysates were centrifuged at 10,000 × *g* at 4 °C for 10 min to remove cell debris and then precleared with protein G Dynabeads (Life Technologies) at 4 °C for 60 min. An input sample of each lysate was set aside for subsequent analysis. Each lysate was divided and incubated with 5 μg/ml of α-AGO2 (Abnova) or mouse IgG (Millipore) on a rotator overnight at 4 °C. Complexes were pulled down by incubating with protein G Dynabeads on a rotator for 1 h at 4 °C. Beads were washed as follows: two times with lysis buffer; three times with lysis buffer with 900 mm NaCl and 1% Nonidet P-40; and three times with lysis buffer. Washed beads and input samples were treated with DNA digestion solution (40 mm Tris-HCl, pH 8.0, 10 mm MgSO_4_, 1 mm CaCl_2_, 200 units/ml RNasin, and 0.04 units/ml DNase I (Promega)) at 37 °C for 20 min.

##### qRT-PCR

Total RNA was extracted from cells or protein A or G beads with TRIzol (Life Technologies). Reverse transcription was performed using the Superscript III First-Strand Synthesis SuperMix for qRT-PCR Kit (catalog number 11752-050, Life Technologies) for mRNA quantitation or the TaqMan microRNA RT Kit (catalog number 4366596, Life Technologies) for miRNA quantitation. PCR was performed in triplicate on an ABI 7900HT (Applied Biosystems) using the following TaqMan expression assays (Life Technologies): miR-1, 000385; miR-206, 000510; miR-133b, 002247; Igf-1, Mm00439560_m1; and HDAC4, Mm01299558-g1, and was analyzed with SDS software (Life Technologies).

##### Western Analyses and ELISAs

Flash-frozen mouse skeletal muscle samples (quadriceps femoris) were homogenized using a Bullet Blender (Next Advance) in radioimmunoprecipitation assay buffer containing protease inhibitors, and protein was quantitated using a Micro BCA protein assay kit (Thermo Scientific, 23235). For Western analyses, 20 μg of each sample was diluted 1:1 in Laemmli buffer and run on 4–20% gradient SDS-PAGE gels (Bio-Rad). After transfer to Immobilon-FL (Millipore) membrane and blocking in Odyssey blocking buffer (LI-COR), blots were probed with the following antibodies: rabbit monoclonal α-HDAC4 (Cell Signaling, catalog number 2072; 1:1000), rabbit polyclonal α-TDP-43 (Proteintech, catalog number 10782-2-AP; 1:500), or mouse monoclonal α-GAPDH (Abcam, ab8245; 1:5000) diluted in Odyssey blocking buffer and 0.01% Tween 20 overnight at 4 °C. Blots were washed in PBST (PBS, 0.05% Tween 20) and incubated in the appropriate IRDye-conjugated secondary antibody (LI-COR) for 1 h at room temperature, washed in PBST, and imaged and quantitated on a LI-COR Odyssey system. For ELISAs, samples were diluted to 1 μg/μl and assayed in triplicate using the IGF-1 mouse ELISA kit (Abcam, ab100695) according to the manufacturer's instructions. Colorimetric signal was measured using a Victor 1420 multilabel counter (PerkinElmer).

## RESULTS

### 

#### 

##### The miR-1 Family Physically Interacts with TDP-43

To identify proteins that physically interact with and might regulate activity of the *miR-1/miR-206* family, but not the *miR-133* family (Fig. 1*A*), we performed RNA electrophoretic mobility shift assays (EMSAs) seeking proteins that uniquely bind and alter the migration of these miRNAs. We used fluorescently labeled mature *miR-1* and protein lysates from undifferentiated C_2_C_12_ cells, a mouse skeletal myoblast cell line. We found a prominent band representing an miRNA-protein complex in C_2_C_12_ lysates incubated with labeled *miR-1* that was effectively lost with the addition of excess unlabeled *miR-1* or *miR-206*, but not with unlabeled *miR-133* (Fig. 1*B*). The same band was observed when fluorescently labeled *miR-206* was incubated with C_2_C_12_ lysates and could be competed with either *miR-1* family member, but not with *miR-133* (Fig. 1*B*). We concluded that a protein or complex of proteins in C_2_C_12_ cells preferentially interacts with the mature form of the *miR-1/miR-206* family, but not *miR-133*, *in vitro*.

To determine the identity of the proteins that interacted with the *miR-1* family in EMSAs, we used biotinylated mature *miR-1* to affinity-purify the interacting proteins. To ensure that biotinylated *miR-1* could be used as a bait, we confirmed that biotinylating *miR-1* did not affect its ability to compete with labeled *miR-1* for interaction with the protein complex (Fig. 1*B*). Proteins that co-precipitated with biotinylated *miR-1* were separated on denaturing polyacrylamide gels (Fig. 1*C*), and mass spectrometry was used to identify the bands that emerged in *miR-1*, but not control or *miR-133a*, pulldowns. The major proteins identified were KSRP and TDP-43. Both of these RNA-binding proteins physically interact with components of the microprocessor or RISC (18, 24). Although KSRP is required for biogenesis of the *miR-1* family in specific contexts (18), the effects of TDP-43 on the *miR-1* family have not been reported.

To test whether the interaction of *miR-1/miR-206* with TDP-43 occurs in a cellular context, we performed CLIP of TDP-43 from undifferentiated C_2_C_12_ cells, which express *miR-206* and *miR-133b*, but not abundant *miR-1* or *miR-133a* (Fig. 1*D*). Despite cross-linking, qRT-PCR of the co-precipitated RNA showed that *miR-133b* was not recovered from the TDP-43 CLIP (Fig. 1*E*). In contrast, *miR-206* was immunoprecipitated with TDP-43 in myoblasts (Fig. 1*E*). These results confirm that the TDP-43-*miR-1* family interaction occurs endogenously in muscle cells.

##### Loss of TDP-43 Enhances miR-1 Effects in Drosophila

**FIGURE 2.**
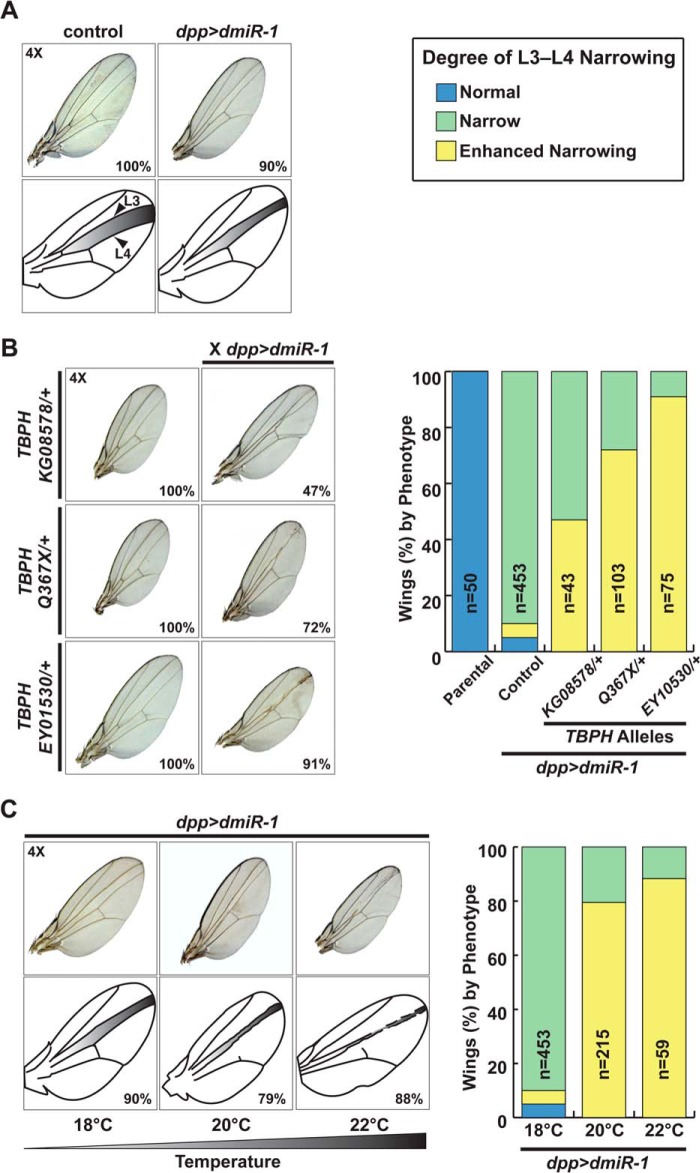
**Loss of *TBPH*, the *Drosophila Tdp-43* ortholog, increases *miR-1* activity in fly wings.** *A*, wings from control or *dppGAL4::UAS-dmiR-1*-expressing flies (*dpp*>*dmiR-1*) show that *miR-1* expression causes decreased L3-L4 intervein distance (25). *B*, fly wings from intercrosses of three different *TBPH* hypomorphic lines to *dpp*>*dmiR-1* flies showed enhancement of the *dpp*>*dmiR-1* phenotype. Quantitation of wing phenotypes from parental, *dpp*>*dmiR-1* (*Control*), or *dpp*>*dmiR*-1 X *TBPH* hypomorph crosses is shown. *C*, wing morphology in flies carrying the temperature-sensitive *dpp*>*dmiR-1* allele housed at 18, 20, or 22 °C. Quantitation of wing phenotypes is shown. *Narrow* is defined as L3-L4 intervein distance of *dpp*>*dmiR-1* at 18 °C; *Enhanced Narrowing* is defined as ≥50% further reduction in L3-L4 intervein distance as compared with *dpp*>*dmiR-1* at 18 °C.

To validate the interaction between TDP-43 and *miR-1* in a more *in vivo* context, and to determine the consequences on *miR-1* function, we turned to the *Drosophila* system. In *Drosophila* wings, misexpression of *Drosophila miR-1* (*dmiR-1*) under the control of a *decapentaplegic* (*dpp*) promoter leads to decreased long vein 3-4 (L3-L4) intervein distance (Fig. 2*A*). This system has been used to identify proteins that genetically interact with *dmiR-1* by scoring for the loss or gain of intervein distance in wings of offspring generated from crosses to mutant lines (25). We obtained three fly lines harboring hypomorphic or null alleles of *TBPH*, the *Drosophila* ortholog of *Tdp-43*, which each had normal wing morphology in the heterozygous state. When crossed to the *dpp-GAL4::UAS-dmiR-1* (*dpp*>*dmiR-1*) fly line, resulting offspring also heterozygous for *TBPH* mutants had an enhanced narrowing of L3-L4 intervein distance (Fig. 2*B*). Although penetrance varied among the three *TBPH* alleles, the consistently enhanced wing phenotype indicated that the effect was due to loss of Tdp-43 and not to background or allele-specific effects. Furthermore, the combination of reduced Tdp-43 levels and misexpression of *dmiR-1* in the fly wing phenocopied the effects of even higher levels of *dmiR-1* transgene expression induced by a temperature-responsive *dpp*>*dmiR-1* allele at 22 °C (Fig. 2*C*). These experiments reveal that a conserved interaction between *miR-1* and TDP-43 occurs *in vivo*. Furthermore, the fact that reduced Tdp-43 dosage enhanced the effects of *dmiR-1* expression in the fly wing suggests that Tdp-43 may dampen *miR-1* activity.

##### Loss of TDP-43 Increases miR-1/miR-206 Family Activity in Skeletal Myoblasts

**FIGURE 3.**
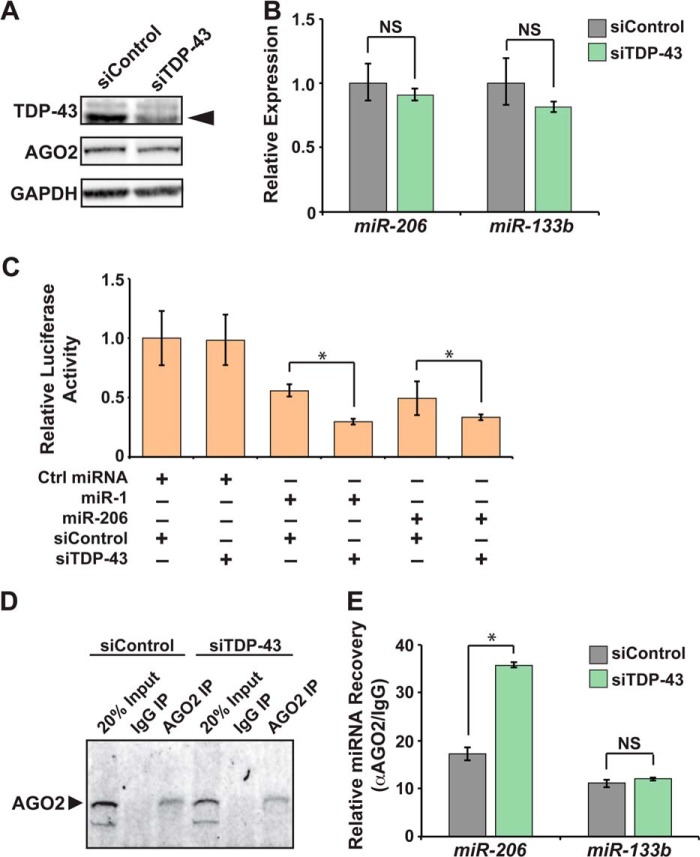
**TDP-43 suppresses activity of *miR-1* and *miR-206* by inhibiting their association with AGO2.** *A*, Western blots to detect TDP-43 and AGO2 in C_2_C_12_ cells transfected with nontargeting control siRNA (*siControl*) or siRNA directed against *Tdp-43* (*siTDP-43*). *Arrow* indicates 43-kDa band corresponding to TDP-43. GAPDH levels show equal protein loading. *B*, *miR-206* and *miR-133b* levels in C_2_C_12_ cells transfected with nontargeting control siRNA (*siControl*) or siRNA directed against *Tdp-43* (*siTDP-43*) as detected by qRT-PCR and normalized to U6 levels. *C*, luciferase reporter assays measuring activity of *miR-1* or *miR-206* in control (*siControl*) or TDP-43-depleted (*siTDP-43*) cells. *Ctrl miRNA*, control miRNA. *D*, Western analysis detecting AGO2 protein following immunoprecipitation from TDP-43-depleted (*siTDP-43*) or control (*siControl*) C_2_C_12_ cells using α-AGO2 (*AGO2 IP*), mouse IgG (*IgG IP*), or 20% input showed efficient and specific recovery of AGO2 protein. *E*, miRNA levels recovered by AGO2 RNA IP from C_2_C_12_ cells transfected with an siRNA targeting *Tdp-43* (*siTDP-43*) were compared with those transfected with a nontargeting control siRNA (*siControl*) (*NS*, not significant; *, *p* < 0.05).

To directly test whether *miR-1/miR-206* activity was suppressed by TDP-43, we returned to C_2_C_12_ skeletal myoblasts where we depleted TDP-43 using a targeted siRNA. Efficient TDP-43 knockdown was confirmed by Western analysis (Fig. 3*A*). Levels of mature *miR-206* and *miR-133b*, which are the most abundant family members expressed in skeletal myoblasts, were comparable in control and TDP-43-depleted cells (Fig. 3*B*), indicating that loss of TDP-43 did not affect expression or biogenesis of these miRNAs. We used these conditions to measure activity of the *miR-1/miR-206* family in luciferase assays. In the presence of TDP-43, *miR-1* was able to repress expression of a luciferase reporter with a validated *miR-1* family binding site in the 3′-UTR (4). Depleting TDP-43 increased *miR-1* repression of this reporter (Fig. 3*C*) without affecting baseline luciferase expression in the presence of a nontargeting control miRNA. TDP-43 similarly enhanced *miR-206*-mediated reporter repression (Fig. 3*C*), demonstrating that loss of TDP-43 enhances activity of both *miR-1* family members.

##### TDP-43 Disrupts Association of miR-1/miR-206 with AGO2

Because miRNA activity requires interaction with the RISC, one potential mechanism by which TDP-43 negatively regulates *miR-1* family activity may be through inhibiting their RISC association, thus decreasing their availability to repress target mRNA sequences. To test this hypothesis, we immunoprecipitated the major RISC component, AGO2, from control or TDP-43-depleted C_2_C_12_ cells (Fig. 3*D*). Western analysis confirmed that TDP-43 depletion did not affect AGO2 expression (Fig. 3*A*). qRT-PCR of the co-precipitated RNA showed that TDP-43 depletion increased the amount of *miR-206* associated with AGO2 without affecting the levels of co-precipitated *miR-133b* (Fig. 3*E*). Thus, loss of TDP-43 enhanced *miR-206* incorporation into the RISC.

##### TDP-43 Overexpression Decreases miR-1/miR-206 Family Activity in Transgenic Mice

**FIGURE 4.**
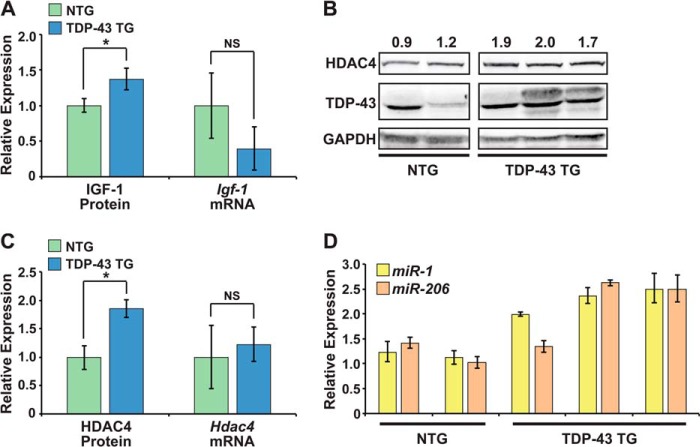
**TDP-43 overexpression decreases *miR-1* family activity in mouse muscle.** *A*, average levels of IGF-1 protein, determined by ELISA, and *Igf-1* mRNA, detected by qRT-PCR, in hindlimb skeletal muscle of 3.5 week old, male, nontransgenic (NTG, *n* = 2) or TDP-43 transgenic (TDP-43 TG, *n* = 3) littermates. *B*, Western blots to detect HDAC4 and TDP-43 protein in hindlimb skeletal muscle of 3.5 week old, male, nontransgenic (NTG) or TDP-43 transgenic (TDP-43 TG) littermates. Relative HDAC4 quantity, as determined by densitometry, is shown above. GAPDH levels show equal protein loading. *C*, average levels of HDAC4 protein and *Hdac4* mRNA in samples analyzed in *A* and *B. D*, relative levels of *miR-1* and *miR-206* detected by qRT-PCR using the same samples analyzed in *A–C* (*NS*, not significant; *, *p* < 0.05).

Multiple TDP-43 transgenic mouse lines have been generated to model ALS. We took advantage of an existing transgenic mouse line with human TDP-43 overexpressed primarily in skeletal muscle to analyze the effects on endogenous *miR-1* family activity *in vivo* (26). Overexpression of the human TDP-43 transgene in muscle results in variable levels of both high and low molecular weight TDP-43 species (Fig. 4*B*) (26). *Igf-1* and *Hdac4* are reported targets of the *miR-1* family in skeletal muscle, and *miR-1* modulation affects protein levels of these two targets, without altering their mRNA expression (5, 27). Using ELISA, we examined protein levels of IGF-1 in skeletal muscle from 3.5-week-old transgenic (TDP-43 TG) and nontransgenic (NTG) littermates, before the onset of muscle weakness (Fig. 4*A*). We found that transgenic mice had higher protein levels of both TDP-43 and the *miR-1* family target, IGF-1 (Fig. 4, *A* and *B*). However, IGF-1 protein tended to be elevated in the muscle of transgenic mice without a corresponding increase in *Igf-1* mRNA (Fig. 4*A*). This type of discordant mRNA and protein expression can be a feature of altered miRNA function and is consistent with a model where TDP-43 limits the activity of the *miR-1/miR-206* family in skeletal muscle, leading to increased IGF-1 translation.

We performed Western analyses to compare levels of a second *miR-1* family target, HDAC4, in the same skeletal muscle preparations in which we measured TDP-43 and IGF-1 (Fig. 4*B*). Similarly, HDAC4 protein was elevated in TDP-43 TG skeletal muscle without an increase in *Hdac4* mRNA (Fig. 4*C*). Thus, two well validated *miR-1* family targets were up-regulated at the protein, but not mRNA level in TDP-43 transgenic muscle, in agreement with our model of reduced *miR-1/206* activity. Elevated HDAC4 may be particularly relevant to the progressive muscle weakness observed in these TDP-43 transgenic mice as HDAC4 is an established negative regulator of muscle innervation (9). Indeed, the reinnervation defect observed in muscle of *miR-206*-null mice was attributed to up-regulation of this critical *miR-1* family target (9).

Interestingly, this apparent decrease in *miR-1* family activity occurred despite elevated levels of both *miR-1* and *miR-206* in TDP-43 TG muscle (Fig. 4*D*), further highlighting the dampening effect that TDP-43 has on *miR-1* family activity in muscle. Together, these results support the idea that *miR-1* family activity is decreased in TDP-43 transgenic muscle, resulting in increased translation of *miR-1* family targets.

## DISCUSSION

Here we show a unique physical and genetic interaction between TDP-43, an ALS disease protein, and the *miR-1* family of muscle miRNAs that negatively regulates *miR-1* family activity. TDP-43 decreased activity of mature *miR-1* and *miR-206*, but not the co-transcribed *miR-133* family, by preventing the bound miRNAs from associating with the RISC. Consequently, TDP-43 overexpression in skeletal muscle led to increased protein levels of the *miR-1* family targets, IGF-1 and HDAC4. This increase was observed despite unchanged *Igf-1* and *Hdac4* mRNA expression and elevated *miR-1* family levels. To our knowledge, a selective mature miRNA-protein interaction that limits miRNA activity, independent of miRNA biogenesis, has not been reported and suggests that the differential activity of mature miRNAs, including bicistronically encoded miRNAs, such as *miR-1* and *miR-133*, can be regulated by selective interaction with RNA-binding proteins.

The predilection of TDP-43 for *miR-1/miR-206* was observed in both *in vitro* miRNA pulldowns as well as *in vivo* CLIP experiments. Furthermore, TDP-43 depletion in myoblasts led to increased interaction of AGO2 with *miR-206*, but not *miR-133b*. TDP-43 overexpression *in vivo* up-regulated IGF-1 and HDAC4, proteins whose synthesis is normally repressed by the *miR-1* family (5, 27). The extent to which TDP-43 affects activity of other miRNAs must be determined, but our results reveal a unique mechanism that may modulate the activity of mature miRNAs, independent of miRNA transcription or biogenesis. Because the *miR-1* family promotes differentiation and the *miR-133* family keeps muscle in a less mature, more proliferative state (5, 12, 13), the TDP-43-*miR-1* family interaction may be important to control the balance of these co-transcribed miRNA families to promote development and maintain adult muscle homeostasis.

*miR-206* expression increases in response to muscle denervation (9). However, the elevated *miR-206* that we observed in transgenic muscle is unlikely to be a response to motor neuron death because these young animals had not yet become symptomatic. Furthermore, *miR-1* levels were elevated in TDP-43 transgenic muscle, although *miR-1* transcription is not affected by denervation (9). Instead, it is possible that interaction with TDP-43 stabilizes *miR-1* and *miR-206*, preventing their degradation or clearance, and leading to their accumulation, while also limiting their RISC-associated activity. This is consistent with the observation that *miR-1* and *miR-206* levels greatly exceed those of *miR-133* in mature muscle (20, 21). Further studies are required to assess the effect of TDP-43 on miRNA stability and turnover.

Given that TDP-43 is an ALS disease protein (28) and deleting *miR-206* in mice exacerbates the phenotype of an ALS mouse model (9), we aimed to elucidate the consequence of the TDP-43-*miR-1* family interaction in skeletal muscle. Dysregulation and aggregation of TDP-43 are commonly observed in motor neurons of ALS subjects, regardless of genetic etiology, but have not been described in skeletal muscle. Considering the ubiquitous expression of TDP-43 and other known genetic ALS determinants, some occurrences of ALS might be accompanied by skeletal muscle TDP-43 abnormalities. This could decrease the activity of the *miR-1* family in affected muscle, thus altering retrograde signaling at the NMJ through dysregulation of both HDAC4 (9) and MEF-2 (14), and ultimately contributing to motor neuron demise. With our finding that TDP-43 overexpression increases protein levels of the *miR-1*/*miR-206* target, HDAC4, whose dysregulation correlates with ALS disease progression (15), further interrogation of TDP-43 levels, localization, and activity in skeletal muscle of individuals with ALS at various stages of the disease seems warranted.

## Footnotes

2 The abbreviations used are:

miRNA: microRNA
miR: microRNA
RISC: RNA-induced silencing complex
NMJ: neuromuscular junction
ALS: amyotrophic lateral sclerosis
KSRP: KH-type splicing regulatory protein
CLIP: cross-linking and immunoprecipitation
IP: immunoprecipitation
qRT-PCR: quantitative RT-PCR
L: long vein
TG: transgenic
NTG: nontransgenic.
